# Below ground efficiency of a parasitic wasp for *Drosophila suzukii* biocontrol in different soil types

**DOI:** 10.1038/s41598-022-12993-w

**Published:** 2022-06-01

**Authors:** Benedikt J. M. Häussling, Melinda Mautner, Johannes Stökl

**Affiliations:** Department of Evolutionary Animal Ecology, Bayreuth University, Bayreuth, Germany

**Keywords:** Agroecology, Invasive species, Agroecology, Invasive species

## Abstract

The parasitoid wasp *Trichopria drosophilae* is promising as a biocontrol agent for controlling the ubiquitous pest *Drosophila suzukii* (Matsumura). Crucial for the successful implementation of any biocontrol agent is a high parasitisation rate by the parasitoid. Most studies investigating the parasitisation rate of *D. suzukii* pupae have focused on parasitisation in the fruit or in a petri dish. However, the predominant pupation site of *D. suzukii* in the field is the soil. Unfortunately, little is known on how well parasitoid wasps can detect and parasitise pupae of *D. suzukii* buried in the soil. Therefore, we conducted soil parasitisation experiments of *T. drosophilae* on *D. suzukii* pupae using two pupation depths in three different soil types (loamy sand, loam, and clay). In all three soil types, we found generally low *D. suzukii* pupae parasitisation rate by *T. drosophilae*, independent of the pupation depth. The pupation behaviour of *D. suzukii* and the parasitisation behaviour of *T. drosophilae* are discussed in detail. For pest control in most soil types, our results mean that the number of *D. suzukii* larvae pupating in the soil should be reduced, e.g., by adding a layer of sandy soil or covering the soil with plastic mulch. This might increase the probability of success when using *T. drosophilae* as a biocontrol agent.

## Introduction

The cosmopolitan fruit pest *Drosophila suzukii*, also called spotted wing drosophila, is still a major challenge for farmers worldwide. Especially in years with favourable conditions for the pest, the risk of total yield losses can be high^1,2^. Therefore, functional integrated pest management methods are essential to control the pest^3,4^. Consequently, extensive knowledge is required for a broad range of different possible control methods. One promising candidate is larval and pupal parasitoids^5–9^. Parasitoids, mostly wasps, lay their eggs in or on a host, for example in the larvae or pupae of *D. suzukii*. The larvae of the parasitoid then feed on the host and eventually kill it.

One advantage of parasitoids as pest control is that they can be mass-reared and released at a certain date. Thus, population growth can be controlled if release is early in the growing season^9^. Especially for *D. suzukii*, early control is necessary because its population can be high in the surrounding habitats and the insects thus mass invade the fruits when they are nearly ripe^10^. In field and laboratory studies, naturally occurring parasitoids, such as the pupal parasitoids *Pachycrepoideus vindemiae* and, especially, *Trichopria drosophilae*, have proved promising results in controlling *D. suzukii*^8,11–15^. The pupal parasitoid *T. drosophilae* can parasitise the pupae of *D. suzukii* during the entire pupal development time^16^. A crucial ability of the parasitoid during parasitisation is locating the host pupae. The location of the pupae of *D. suzukii* can be directly in the fruit^12^, but especially in the field, the large majority of the larvae pupate in the soil underneath the fruit plant^17,18^. This location means that the parasitoid needs to be able to locate the pupae in or near the fruit and in the soil matrix.

Guillén, et al.^19^ found that *P. vindemiae* could only locate pupae of the Mexican fruit fly *Anastrepha ludens* when the pupae were on the soil surface. They could not locate them when they were in the soil. In contrast, another study found that *P. vindemiae* and *T. drosophilae* can parasite *D. suzukii* pupae in the soil^12^. In their study, Wang, et al.^12^ studied the parasitisation rate of *D. suzukii* pupae in fruits and the soil. However, it is unclear whether the pupae were actually buried in the soil or lay accessible on the soil surface. Furthermore, neither Guillén, et al.^19^ nor Wang, et al.^12^ studied the parasitisation rate in different soil types. Therefore, it is still unclear in what soil type and to which soil depth *T.* *drosophilae* is capable of finding and parasitising the pupae of *D. suzukii*.

To answer this question, we investigated the pupation behaviour of *D. suzukii* and the parasitisation rate of *T. drosophilae* in three different standardised soil types (loamy sand, loam, and clay) with the same soil moisture and at two soil depths (0–6 mm and 7–12 mm). Furthermore, the hatching rate of *D. suzukii* was assessed under these soil conditions.

## Material and methods

### Insects

*Drosophila suzukii* were caught in the state of Hesse, Germany, in 2016. The parasitoid wasps *T. drosophilae* were provided by Bioplanet s.r.l. (Cesena, Italy). *D. melanogaster* was the host for the *T. drosophilae*. The *D. suzukii*, *D. melanogaster* and *T. drosophilae* were reared and kept under the conditions as described in Häussling, et al.^15^.

### Standard soil types

Three different standard soils were used. The soils were chosen to be distinctly different in particle size distribution. According to the United States Department of Agriculture (USDA) classification, the soil types used for these experiments were: loamy sand, loam, and clay (Supplementary Table 3). Using these soil types ensured different physical properties for the fly larvae when they pupated and for the wasp when they searched and parasitised pupae in the soil. Soils were obtained from the “Landwirtschaftliche Untersuchungs- und Forschungsanstalt” (LUFA) in Speyer, Germany.

The Water Holding Capacity (WHC) of all soil types were measured (dried at 105 °C), and then the soils were then adjusted to 40% of the maximal WHC for each soil type. This percentage was the optimal soil moisture for finding the pupae in the soil after the parasitisation exposure. In soil with higher soil moistures, finding the pupae in the soil was challenging.

### Experimental set-up

Plastic boxes (135 mm × 80 mm × 120 mm) were used as arenas for the parasitisation. The bottom of the box was filled with soil to 3 cm and covered with a plastic net with 3 mm mesh size. This layer was included to decrease temperature effects from the bottom of the boxes. Above the net, a layer of 6 mm of one of the three standard soils was added, then one more plastic net and 6 mm of the top layer of the same standard soil. The box was closed with a fine mesh net secured by a rubber band on the top of the plastic box. The top had a hole in the middle to allow airflow while the mesh prevented the escape of the flies and wasps.

We tested the parasitisation and pupation rates in the three soil types by providing each experimental box with 50 *D. suzukii* larvae of the 3^rd^ instar that could decide freely in which of the two soil depths they pupated. To test whether the time of the parasitisation influenced the parasitisation rate, we either directly added five female wasps to the larvae or waited for 24 h before releasing the wasps, allowing the larvae to pupate first. The wasps had 24 h to parasitise the pupae in the soil. We did n = 57 replicates, 19 for each soil type, with 50 *D. suzukii* each. To determine the pupation rates without parasitisation, we performed a negative control treatment (n = 39, 13 for each soil type, with 50 *D. suzukii* each). Here, no wasps were released in the boxes. As a positive control, we also analysed the parasitisation rate when all pupae were easily accessible and not buried in the soil. For this positive control (n = 5), 30 pupae of *D. suzukii* were offered on a wet filter paper in a Petri dish to three female *T. drosophilae*. In all treatments, the wasps were provided with a drop of diluted honey and the boxes were placed in a greenhouse. The temperature and the humidity were logged during the investigation.

The pupae of each soil depth and type were collected separately after 24 h of parasitisation time. This time was observed to be sufficient for a successful parasitisation of *D. suzukii*^11,15,16,20,21^. The pupae were photographed next to a precise ruler for scale to determine the pupal size. The length and width of each pupa were measured with the software ImageJ, and the volume was calculated with the formulae from Otto and Mackauer^22^. Afterwards, the pupae were stored in a 96-well-plate in a light-controlled chamber at 24 °C and 70% to 80% RH with a 16:8 h day to night rhythm. The sex of the emerged *D. suzukii* and *T. drosophilae* was recorded.

### Statistical analysis

The effect of the soil type, the soil depth and the presence of a wasp on the proportion of hatched *D. suzukii* was analysed using a binomial generalised linear mixed model (GLMMs) in the R package ‘lme4’^23^. The GLMMs were also used to analyse the effect of soil type and pupation depth on the proportion of hatched *T. drosophilae* and of soil type on *D. suzukii* larvae pupated in the upper soil layer. In both analyses, we added the pupae from each box as a nested random effect. As the interactions among the predictors in the GLMMs were not significant, they were excluded from further analyses. We also found no influence of the time of wasp release and therefore did not differentiate between the two time points in our analyses (see Supplementary Tables 1, 2). Data were analysed in R 3.6.1^24^.

## Results

### Pupation depth of *Drosophila suzukii* larvae

**Figure 1:**
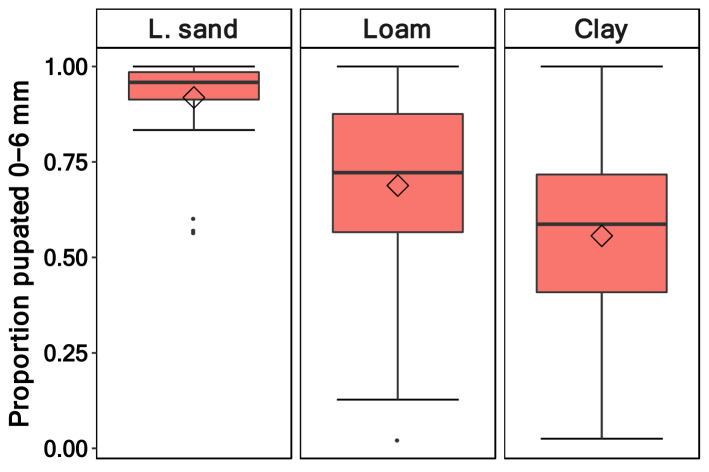
Proportion of *Drosophila suzukii* pupae that pupated in the upper layer (0–6 mm) in relation to the lower layer (7–12 mm). The larvae pupated in the three different soil types: loamy sand, loam and clay (n = 96).

Pupation on the soil surface was an exception, so include them in the pupation depth 0–6 mm. The larvae of *D. suzukii* differed in their pupation depth depending on the soil types (*p* =  < 0.001, Table 1, Fig. 1). In sandy soils, nearly all (median: 96%) pupae pupated in the upper soil layer; in loam soils, the median was 72%, and in clay, the median in the upper layer was 58%. The pupation depth in the sandy soil was significantly different from that in loam and clay soil types. However, the result in the latter two did not differ (Table 2). Additionally, we found that the pupae volume did not affect the pupation depth (*p* = 0.75, Table 1).

**Table 1:** Pupation depth—generalised linear mixed effect model (family = binomial, link = logit, random factors: “Box/pupae volume”, “mean temperature”) examining the effect of soil type and pupal volume on the proportion of larvae (*D.* *suzukii*) pupating in the upper soil layer.

Predictor	χ^*2*^	*df*	*p*-value
Soil type	61.06	2	**< 0.001**
Pupal volume	0.11	1	0.75

Significant values are in bold.

**Table 2:** Multiple comparison between soil types of the pupated pupae. Test: Tukey Honest Significant Difference.

Multiple comparison	Estimate	SE	*Z*	*p*-value
l. sand–loam	2.23	0.39	5.70	**< 0.001**
clay–loam	− 0.76	0.36	− 2.08	0.10
clay–l. sand	− 2.98	0.39	− 7.63	**< 0.001**

Significant values are in bold.

### Hatching rate of *Trichopria drosophilae*

**Figure 2:**
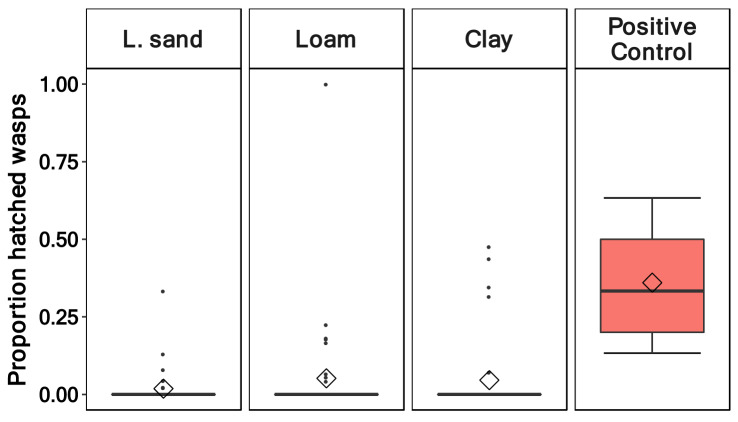
Proportion of hatched *Trichopria drosophilae* wasps out of pupae of *Drosophila suzukii* in the three soil types (loamy sand, loam, and clay) and the positive control where the wasp had free access to the pupae in a petri dish. (Soil types: n = 57, positive control: n = 5).

**Figure 3:**
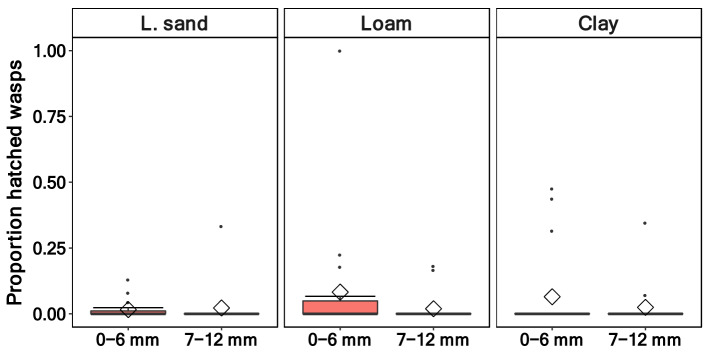
Proportion of hatched *Trichopria drosophilae* wasps between two different pupation depths (0–6 mm and 7–12 mm) in the soil types loamy sand, loam and clay (n = 57).

The proportion of emerged *T. drosophilae* was low for all soil types and pupation depths (Fig. 2). The soil type (*p* = 0.72) and the pupation depth (*p* = 0.11) had no effect on the proportion of emerged wasps (Table 3). In loamy sand, wasps hatched, on average, out of 1.8% of the pupae, followed by clay with 4.5% and loam with 5.1% (Fig. 2). In contrast, in the positive control, where the wasps had free access to the pupae in a petri dish, the mean hatching rate was much higher (36%). The wasp hatching rate of the two pupation depths was similar for loamy sand (0–6 mm 1.5%; 7–12 mm 2.2%). In loam (0–6 mm 8.3%; 7–12 mm 1.9%) and clay soil (0–6 mm 6.5%; 7–12 mm 2.5%, Fig. 3), the difference was more distinct but also not statistically significant.

**Table 3:** Hatched wasps—generalised linear mixed effect model (family = binomial, link = logit, random factors: “Box/pupae number”, “Percent Pupated”) examining the effects of pupation depth and soil type on the hatching rate of the wasp *T. drosophilae* on *D. suzukii* pupae.

Predictor	χ^*2*^	*df*	*p*-value
Soil type	0.68	2	0.71
Pupation depth	2.53	1	0.11

### Hatching rate of *Drosophila suzukii*

**Figure 4:**
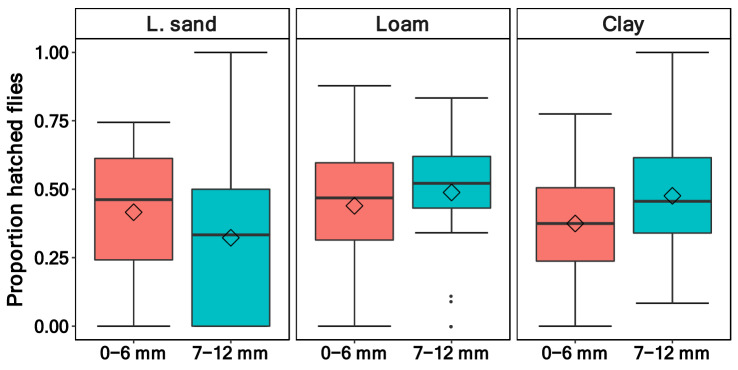
Proportion of hatched *Drosophila* *suzukii* flies between the two pupation depths 0–6 mm and 7–12 mm and the three soil types (loamy sand, loam and clay; n = 96 as waps and control treatments showed no difference).

**Figure 5:**
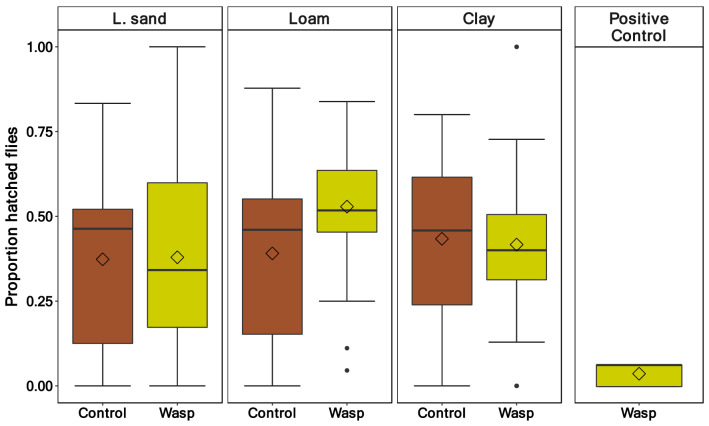
Proportion of hatched *Drosophila* *suzukii* flies from the three soil types (loamy sand, loam, and clay). Pupae were either exposed to the parasitoid wasp (Wasp) or not (Control). The fourth panel displays the number of hatched *D.* *suzukii* in the positive control, where the wasps had free access to the pupae in a petri dish (wasp: n = 57, control: n = 39, positive control: n = 5).

There was no difference in the hatching rates of *D. suzukii* between the negative control and the wasp treatment (*p* = 0.11, Table 4). Furthermore, neither the proportion of emerged *T. drosophilae* nor the soil type (*p* = 0.08) affected the emergence rates of *D. suzukii*. Only the pupation depth affected the proportion of hatched flies (*p* = 0.005), with more flies hatching out in the deeper soil layer (*p* = 0.02, Table 5, Fig. 4). The median of the hatching rate was between 34.2 and 51.4% (Fig. 5). In contrast, in the positive control, in which the wasps had free access to the pupae, the hatching rate of the flies was much lower, with a median of 6.7%.

**Table 4:** Hatched flies—generalised linear mixed effect model (family = binomial, link = logit, random factors: “Box/Pupae number”, “Percent pupated”, “Mean Temperature”) output quantifying the effect of pupation depth, soil type and the treatment (with wasp/without wasp) on the hatching rate of the fly *D.* *suzukii.*

Predictor	χ^*2*^	*df*	*p*-value
Soil type	5.10	2	0.08
Pupation depth	7.87	1	**0.005**
Treatment	2.51	1	0.11

Significant values are in bold.

**Table 5:** Multiple comparison between pupation depth of the hatched flies. Test: Tukey Honest Significant Difference.

Multiple comparison	Estimate	SE	*Z*	*p*-value
7-12–0-6 mm	0.35	0.13	2.81	**0.02**

Significant values are in bold.

## Discussion

The pupae of *D. suzukii* buried in soils were rarely parasitised by the wasp *T. drosophilae* in all three soil types, with wasps emerging from only 1.8% to 5.1% of the fly pupae. This result clearly demonstrates that the parasitisation of host pupae in soils through *T. drosophilae* is an exception. The few hatched wasps were mainly from the upper soil layer. When the wasp had free access in the positive control, the percentage of emerged wasps raised to 36%. This range was also observed from Chabert, et al.^7^.

These findings are consistent with the earlier results of Guillén, et al.^19^, who observed very low parasitisation of pupae of the Mexican fruit fly *Anastrepha ludens* by the pupal parasitoid *Pachycrepoideus vindemiae* in the soil. In fact, in their study, the parasitisation of pupae only happened on the soil’s surface. In our experiments, we had nearly no pupation on the top of the soil. Therefore, the few parasitisations in our study mainly happened in the soil. In contrast, Wang, et al.^12^ found high parasitisation rates of *D suzukii* pupae in soils by *T. drosophilae.* (approx. 55%) and *P. vindemiae* (approx. 30%), but they did not differentiate between soil types or pupation depths. Perhaps the pupae were easily accessible on the soil surface in their study.

The low proportion of hatched wasps in our study is probably due to the physical properties of the soil, which hamper the wasps’ ability to pass the soil matrix and parasitise the host pupae. Another possibility is, although it has yet to be verified, that *T. drosophilae* probably recognises its host through kairomones, and the kairomones are reduced when the host pupae are buried in the soil. This reduction is could be caused by the complex medium soil, where semiochemicals do not diffuse to outside the soil^25^.

The number of hatched flies did not differ between the control treatment and the wasp treatment in soils (Fig. 5). In contrast, the number of emerged *T. drosophilae* wasps was seven times higher in the positive control without soil than in the treatment with soil (Fig. 2). Therefore, we conclude that the low number of hatched wasps is due to low parasitisation rates, rather than a difference in the flies’ immune responses between soil treamtents and the control.

Previous studies have shown that the majority of *D. suzukii* larvae pupate in the soil and less often near or in the fruit^17,18^. It seems that the choice of the larvae’s pupation site depends on the interspecific competition of the fly larvae: more larvae pupate outside of the fruit with increasing competition^26^. In our experiment, we found that most larvae, which had only the choice of pupating in the soil, pupated in the upper 0–6 mm soil layer (only a few on the top). We did not expect high interspecific competition in our experiment due to the relatively low number of larvae. The larvae appear to choose their pupation depth differently depending on the soil type. In sandy soils, they pupate almost exclusively in the upper soil layer; in clay and loam soil, pupation also happened in the lower soil layers. Renkema and Devkota^27^ also found that, in the field, the majority of larvae pupate in the upper soil layer, especially in saturated sandy soils. In contrast, in dry sandy soils, most larvae either desiccated or pupated on the soil surface. The pupation behaviour of *D. suzukii* larvae is affected by the soil type. In soils with smaller particles sizes (e.g., clay soil), *D. suzukii* larvae had a deeper pupation depth than in soils with larger particle sizes (Fig. 1, e.g., sandy loam, Supplementary Table 3). A possible explanation could be that the presence of predominantly larger soil particles hampers larvae movement. We observed that, particularly in sandy soils, several soil particles were attached to the pupae, possibly decreasing the movement ability of the larvae. This hypothesis is supported by the fact that in *Bactrocera oleae*, increasing soil particle size was found to reduce pupation depth^28,29^.

Genetic analyses confirmed that a single gene can explain the pupation behaviour and preference for habitat differences (fruit or soil) in *D. melanogaster*^30,31^. The habitat choice of *D. melanogaster* is influenced not only by the soil water content but also by the air temperature and the fly strain. The larvae tend to choose the best suitable pupation site for emergence, which is high soil water content and has optimal temperature of 25 °C^32^. As for *D. melanogaster*, our results show the same effect for *D. suzukii* in which pupation in wet soils (40% WHC) also occurs in the upper soil layer. Furthermore, it is also possible that *D. suzukii* have a genetic polymorphism in their population that determines the pupation site similar to that of *D. melanogaster*. Interestingly, we found a significantly higher hatching rate for *D. suzukii* adults in the deeper soil layer. The higher hatching rates might arise from more favourable temperature conditions in deeper soil depth and/or, that only the physically fitter larvae can move to deeper soil layers.

In conclusion, this study shows that the soil is a massive barrier for the parasitoid *T. drosophilae* when parasitising its host.

Our results can be implemented in an integrated pest management method by adding a layer of sandy soil or a plastic mulch^33^ on top of the ground under the fruit plants. This layer could decrease the hatching rate of *D. suzukii* due to the desiccation of the larvae and would expose the pupae to a range of antagonists, including *T. drosophilae*^18^. The area covered with the sandy soil can be minimal because *D. suzukii* larvae have a limited movement ability of less than 7.5 cm^34^.

## Supplementary Information

Supplementary Information 1.

Supplementary Information 2.

Supplementary Tables.

## Data Availability

All data generated or analysed during this study are included in this published article (and its supplementary information files).
