# Supplementary material for: Below ground efficiency of a parasitic wasp for Drosophila suzukii biocontrol in different soil types
Source: Sci Rep. 2022 Jun 1;12:9130. doi: 10.1038/s41598-022-12993-w (PMC9160073; doi:10.1038/s41598-022-12993-w)
Supplement: Supplementary file 1 — Supplementary Information 1. [file 41598_2022_12993_MOESM1_ESM.pdf]

R Notebook

Basic system setup

Clean up and install/load packages

If not yet installed, pacman will install and load necessary packages.

```
rm(list = ls())
if(!require('pacman'))install.packages('pacman')
```

```
## Loading required package: pacman
```

```
pacman::p_load(readxl,
               lme4,
               glmmTMB, # instead of lme4, when model is overdispersed
               #for negative binomial or beta-binomial
               car,
               ggplot2,
               cowplot,
               stringr,
               MuMIn,
               ggpubr,
               multcomp, # for Tukey
               plyr,
               reshape2,
               blmeco,
               DHARMa, # residual diagnostics for regression models
               emmeans, # posthoc analysies for interactions
               multcompView, # for graphs signifcances letter
               base, # for mean value by groups
)
```

```
##
## Your package installed
```

ETL - extract, transform, load

Extract: read from excel file

```
# place all data from local directory
Daten <-
```

```
  read_excel("Soil_parasitisation.xlsx",
             sheet = "Tabelle2"
             )

Daten_rep <-
  read_excel("Soil_parasitisation.xlsx",
             sheet = "Tabelle3",
             na = "NA")
Daten_temp <-
  read_excel("Soil_parasitisation.xlsx",
             sheet = "Tabelle1",
             na = "NA")
```

Transform subset

```
Daten_rep$Duration <- as.factor(Daten_rep$Duration)
Daten_rep$Soil_type <- as.factor(Daten_rep$Soil_type)
Daten_rep$Pupation_depth <- as.factor(Daten_rep$Pupation_depth)
Daten$Mean_Temperature <- as.numeric(Daten$Mean_Temperature)

#subsets Daten
Daten$Treatment <- as.factor(Daten$Treatment)
#Daten$Mean_Temperature <- as.factor(Daten$Mean_Temperature)
Daten$Soil_type <- as.factor(Daten$Soil_type)
Daten$Pupation_depth <- as.factor(Daten$Pupation_depth)
Daten$Duration <- as.factor(Daten$Duration)
Daten_test <- subset(Daten, Treatment == 'Test')
Daten$Duration <- as.factor(Daten$Duration)

#subsets Daten_rep

subset_rep_0_6mm <- subset(Daten_rep, Pupation_depth == '0-6 mm')
Daten_rep_wc <-
  subset(Daten_rep, Treatment == 'Wasp' | Treatment == 'Control')
Daten_rep_test_pos_cont <-
  subset(Daten_rep, Treatment == 'Wasp' |
           Treatment == 'Positiv Control')
Daten_rep_test <-  subset(Daten_rep, Treatment == 'Wasp')

subset_48h_rep_all <-  subset(Daten_rep, Duration == '48')
subset_48h_test_postivcontrol_rep <-
  subset(subset_48h_rep_all,
         Treatment == 'Wasp' | Treatment == 'Positiv Control')
subset_48h_postivcontrol_rep <-
  subset(subset_48h_rep_all, Treatment == 'Positiv Control')

# Data with temprature for 48h
Daten_temp$Duration <- as.factor(Daten_temp$Duration)
subset_total_temp <- subset(Daten_temp, Treatment == 'Test')
```

Design presets

Some plots share their design, which is set up here

```
theme_wasp <- function() {
  theme_gray() +
    theme(
      plot.title = element_text(
        color = "black",
        face = "bold",
        size = 18,
        hjust = 0.5
      ),
      axis.title = element_text(
        color = "black",
        face = "bold",
        size = 18
      ),
      axis.title.x = element_text(size = 14),
      axis.title.y = element_text(size = 16),
      axis.text.x = element_text(
        face = "bold",
        size = 14,
        color = "black"
      ),
      axis.text.y = element_text(
        face = "bold",
        size = 14,
        colour = "black"
      ),
      strip.background = element_rect(fill = "white", colour = "black"),
      panel.background = element_rect(fill = "white", colour = "black"),
      strip.text = element_text(face = "bold", size = 16),
      axis.text = element_text(face = "bold.italic"),
      panel.grid = element_blank()
    )
}
```

#Test duration ##Fly Supplementary Tabel 1

```
m2 <-
  glmer(
    Hatched_Fly ~ Duration + Soil_type + Pupation_depth + Treatment +
      (1 |Repetition / pupae_number) + (1 |Percent_pupated) +
      (1 | Mean_Temperature),
    data = Daten,
    family = "binomial"
  )
```

```
## boundary (singular) fit: see help('isSingular')
```

```
summary(m2)
Anova(m2, type = "II")
summary(glht(
  m2,
  mcp(
    Soil_type = "Tukey",
    Pupation_depth = "Tukey",
    Treatment = "Tukey",
    Duration = "Tukey"
  )
))
# No multiple way interaction and no 2 way interaction only between treatment
#and mean_temprature but this one does not make sense in biological context
Residuals_calcualte4 <-
  simulateResiduals(fittedModel = m2, plot = T)
```

```
# Duration has no sig. effect on the hatched wasp
```

Wasp Supplementary Tabel 2

```
m2 <-
  glmer(
    Hatched_Wasp ~ Duration + Pupation_depth + Soil_type  +
      (1 |Repetition / pupae_number) + (1 | Percent_pupated),
    data = Daten_test,
    family = "binomial",
    na.action = na.omit
  )
r.squaredGLMM(m2)
summary(m2)
Anova(m2, type = "II")
summary(glht(
  m2,
  mcp(
    Soil_type = "Tukey",
    Pupation_depth = "Tukey",
    Duration = "Tukey"
  )
))
Residuals_calcualte4 <-
  simulateResiduals(fittedModel = m2, plot = T)
```

```
# Temperature had no effect on the hatched wasp and as Random effect
#only very small variance effect so it was excluded
# Duration has no sig. effect on the hatched wasp
```

GLM Hatched Fly

Hatched Fly Tabel 4, 5

```
m2 <-
  glmer(
    Hatched_Fly  ~ Soil_type + Pupation_depth + Treatment +
      (1 |Repetition / pupae_number) +
      (1 |Percent_pupated) + (1 | Mean_Temperature),
    data = Daten,
    family = "binomial",
  )
```

```
## boundary (singular) fit: see help('isSingular')
```

```
summary(m2)
Anova(m2, type = "II")
summary(glht(
  m2,
  mcp(
    Soil_type = "Tukey",
    Pupation_depth = "Tukey",
    Treatment = "Tukey"
  )
))
Residuals_calcualte4 <-
  simulateResiduals(fittedModel = m2, plot = T)
```

GLM Hatched wasp

Hatched Wasps Tabel 3

```
m2 <-
  glmer(
    Hatched_Wasp ~   Soil_type + Pupation_depth +
      (1 |Repetition / pupae_number) + (1 | Percent_pupated),
    data = Daten_test,
    family = "binomial",
    na.action = na.omit,
  )
summary(m2)
Anova(m2, type = "II")
summary(glht(m2, mcp(
  Soil_type = "Tukey", Pupation_depth = "Tukey"
)))
Residuals_calcualte4 <-
  simulateResiduals(fittedModel = m2, plot = T)
```

```r
# Temperature and Duration had no effect on the hatched wasp and as
# Random effect only very small variance effect so it was excluded
```

Pupation depth

Pupation site (upper layer) effect of soil type and pupal volume (Table 1, 2)

```r
m3 <-
  glmer(
    Pupation_depth_factor ~  Soil_type  + Pupaevolume +
      (1 |Repetition / pupae_number) + (1 | Mean_Temperature),
    data = Daten,
    family = "binomial"
    )
```

```
## boundary (singular) fit: see help('isSingular')
```

```r
r.squaredGLMM(m3)
```

```
## boundary (singular) fit: see help('isSingular')
```

```r
summary(m3)
Anova(m3)
summary(glht(m3, mcp(Soil_type = "Tukey")))
Residuals_calcualte4 <-
  simulateResiduals(fittedModel = m3, plot = T)
```

```r
? outliers
```

Graphics

Pupation depth (Figure 1)

```r
means.sem2 <-
  ddply(
    subset_rep_0_6mm,
    c("Soil_type"),
    summarise,
    mean = mean(Percent_pupated, na.rm = T),
    sem = sd(Percent_pupated, na.rm = T) /
      sqrt(length(Percent_pupated[!is.na(Percent_pupated)])),
    median = median(Percent_pupated, na.rm = T)
  )
means.sem3 <-
  transform(means.sem2, lower = mean - sem, upper = mean + sem)
```

```r
#Regroup Soil type
subset_rep_0_6mm_regroup <- subset_rep_0_6mm
subset_rep_0_6mm_regroup$Soil_type <-
  factor(subset_rep_0_6mm_regroup$Soil_type,
         levels = c("Loamy sand", "Loam", "Clay"))
# New facet label names for Soil type
soil_type.names <- c("L. sand", "Loam", "Clay")
names(soil_type.names) <- c("Loamy sand", "Loam", "Clay")

#plot
plot2_pupation_depth <- ggplot(data = subset_rep_0_6mm_regroup) +
  stat_boxplot(
    aes(x = Treatment_pupation_depth, y = Percent_pupated,
        fill = Treatment_pupation_depth),
    geom = "errorbar",
    position = position_dodge(0.9)
  ) +
  geom_boxplot(
    aes(x = Treatment_pupation_depth, y = Percent_pupated,
        fill = Treatment_pupation_depth),
    position = position_dodge(0.9),
    width = 0.8
  ) +
  stat_summary(
    aes(x = Treatment_pupation_depth, y = Percent_pupated,
        fill = Treatment_pupation_depth),
    position = position_dodge(0.9),
    fun.y = mean,
    geom = "point",
    shape = 5,
    size = 4
  ) +
  facet_grid( ~ Soil_type,
             labeller = labeller(Soil_type = soil_type.names)) +
  theme_wasp() +
  ggplot2::theme(
    legend.position = "none",
    axis.title.x = element_blank(),
    axis.text.x = element_blank(),
    axis.ticks.x = element_blank()
  ) +

  ggplot2::labs(y = expression(bold(paste(
    "Proportion pupated 0-6 mm"
  ))))
plot2_pupation_depth
```

```r
pdf("boxplot_pupation_depth.pdf",
    width = 6,
    height = 4)
print(plot2_pupation_depth)
dev.off()
```

Proporition of hatched flies (Figure 4)

```
means.sem2 <-
  ddply(
    Daten_rep_wc,
    c("Soil_type","Treatment"),
    summarise,
    mean = mean(Proportion_of_hatched_flies, na.rm = T),
    sem = sd(Proportion_of_hatched_flies, na.rm = T) /
      sqrt(length(Percent_pupated)),
    median = median(Proportion_of_hatched_flies, na.rm = T)
  )
means.sem3 <-
  transform(means.sem2, lower = mean - sem, upper = mean + sem)
#Regroup Soil type
Daten_rep_wc_regroup <-Daten_rep_wc
Daten_rep_wc_regroup$Soil_type <- factor(Daten_rep_wc_regroup$Soil_type,
                                         levels = c("Loamy sand","Loam", "Clay" ))
# New facet label names for Soil type
soil_type.names <- c("L. sand","Loam", "Clay")
names(soil_type.names) <- c("Loamy sand","Loam", "Clay")
# Plot
plot2_pupation_depth <- ggplot(data = Daten_rep_wc_regroup) +
      stat_boxplot(
        aes(x = Treatment, y = Proportion_of_hatched_flies,fill = Treatment),
        geom = "errorbar",
        position = position_dodge(0.9)
      ) +
      geom_boxplot(
        aes(x = Treatment, y = Proportion_of_hatched_flies,fill = Treatment),
        position = position_dodge(0.9),
        width = 0.8,
      ) +
      stat_summary(
        aes(x = Treatment, y = Proportion_of_hatched_flies, fill = Treatment),
        position = position_dodge(0.9),
        fun.y = mean,
        geom = "point",
        shape = 5,
        size = 4
      ) +
      facet_grid(~Soil_type,
                 labeller = labeller( Soil_type = soil_type.names )) +
      ylim(0, 1) +
      theme_wasp()+
      ggplot2::theme(
      legend.position = "none",
      axis.title.x = element_blank(),
  ) +
      ggplot2::labs(y = expression(bold(paste(
      "Proportion hatched flies"
  ))) )
plot2_pupation_depth2 <- plot2_pupation_depth +
```

```
  scale_fill_manual(values = c("sienna","yellow3"))
plot2_pupation_depth2
```

```
pdf("boxplot_hatched_flies.pdf",
    width = 6,
    height = 4)
print(plot2_pupation_depth2)
dev.off()
```

Positive Control

```
#Regroup Soil type
subset_48h_rep_regroup <- subset_48h_postivcontrol_rep
subset_48h_rep_regroup$Soil_type <-
  factor(subset_48h_rep_regroup$Soil_type,
         levels = c("Positive C."))
# New facet label names for Soil type
soil_type.names <- c("Positive\nControl")
names(soil_type.names) <- c("Positive C.")
# Plot
plot2_postiv_control <- ggplot(data = subset_48h_rep_regroup) +
  stat_boxplot(
    aes(x = Treatment_positiv_control, y = Proportion_of_hatched_flies,
        fill = "#CC3333"),
    geom = "errorbar",
    position = position_dodge(0.9)
  ) +
  geom_boxplot(
    aes(x = Treatment_positiv_control, y = Proportion_of_hatched_flies,
        fill = Treatment_positiv_control),
    position = position_dodge(0.9),
    width = 0.8
  ) +
  stat_summary(
    aes(x = Treatment_positiv_control, y = Proportion_of_hatched_flies,
        fill = Treatment_positiv_control),
    position = position_dodge(0.9),
    fun.y = mean,
    geom = "point",
    shape = 5,
    size = 4
  ) +
  facet_grid( ~ Soil_type,
              labeller = labeller(Soil_type = soil_type.names)) +
  ylim(0, 1) +
  theme_wasp() +
  ggplot2::theme(
    legend.position = "none",
    axis.title.x = element_blank(),
    axis.title.y = element_blank(),
    axis.text.y = element_blank(),
```

```
    axis.ticks.y = element_blank()
  ) +

  ggplot2::labs(y = expression(bold(paste(
    "Proportion hatched flies"
  ))))
plot2_postiv_control
```

```
plot2_postiv_control  <- plot2_postiv_control  +
  scale_fill_manual(values = c("sienna", "yellow3"))
plot2_postiv_control
```

```
print(plot2_postiv_control)
```

```
dev.off()
```

Mulitple plot hatched flies

```
plot_multi_hatched_flies <- plot_grid(
  plot2_pupation_depth2,
  plot2_postiv_control,
  label_size = 18,
  ncol = 2,
  nrow = 1,
  rel_widths = c(1, 0.25)
)
plot_multi_hatched_flies
```

```
pdf("plot_multi_hatched_flies.pdf",
    width = 10,
    height = 6)
print(plot_multi_hatched_flies)
dev.off()
```

Pupation depth to hatched fly (Figure 5)

```
#Regroup Soil type
Daten_rep_wc_regroup <- Daten_rep_wc
Daten_rep_wc_regroup$Soil_type <-
  factor(Daten_rep_wc_regroup$Soil_type,
         levels = c("Loamy sand", "Loam", "Clay"))
# New facet label names for Soil type
soil_type.names <- c("L. sand", "Loam", "Clay")
names(soil_type.names) <- c("Loamy sand", "Loam", "Clay")
# Plot
plot2_pupation_depth <- ggplot(data = Daten_rep_wc_regroup) +
  stat_boxplot(
    aes(x = Pupation_depth, y = Proportion_of_hatched_flies,
```

```
        fill = Pupation_depth),
    geom = "errorbar",
    position = position_dodge(0.9)
  ) +
  geom_boxplot(
    aes(x = Pupation_depth, y = Proportion_of_hatched_flies,
        fill = Pupation_depth),
    position = position_dodge(0.9),
    width = 0.8
  ) +
  stat_summary(
    aes(x = Pupation_depth, y = Proportion_of_hatched_flies,
        fill = Pupation_depth),
    position = position_dodge(0.9),
    fun.y = mean,
    geom = "point",
    shape = 5,
    size = 4
  ) +
  facet_grid( ~ Soil_type,
              labeller = labeller(Soil_type = soil_type.names)) +
  ylim(0, 1) +
  theme_wasp() +
  ggplot2::theme(legend.position = "none",
                 axis.title.x = element_blank())+
  ggplot2::labs(y = expression(bold(paste(
                   "Proportion hatched flies"
                 ))))
                 plot2_pupation_depth
```

```
                 pdf("boxplot_hatched_flies_pupae_depth.pdf",
                     width = 8,
                     height = 4)
                 print(plot2_pupation_depth)
                 dev.off()
```

Porporiton of hatched wasps (Figure 2)

```
means.sem2 <-
  ddply(
    Daten_rep_test_pos_cont,
    c("Soil_type", "Pupation_depth"),
    summarise,
    mean = mean(Prportion_of_Hatched_wasps, na.rm = T),
    sem = sd(Prportion_of_Hatched_wasps, na.rm = T) /
      sqrt(length(Percent_pupated)),
    median = median(Prportion_of_Hatched_wasps, na.rm = T)
  )
means.sem3 <-
  transform(means.sem2, lower = mean - sem, upper = mean + sem)
```

```r
#Regroup Soil type
Daten_rep_test_pos_cont_regroup <- Daten_rep_test_pos_cont
Daten_rep_test_pos_cont_regroup$Soil_type <-
  factor(
    Daten_rep_test_pos_cont_regroup$Soil_type,
    levels = c("Loamy sand", "Loam", "Clay", "Positive C.")
  )

# New facet label names for Soil type
soil_type.names <- c("L. sand", "Loam", "Clay", "Positive\nControl")
names(soil_type.names) <-
  c("Loamy sand", "Loam", "Clay", "Positive C.")

#plot
plot2_pupation_depth48 <-
  ggplot(data = Daten_rep_test_pos_cont_regroup) +
  stat_boxplot(
    aes(x = Treatment_positiv_control, y = Prportion_of_Hatched_wasps,
        fill = Treatment_positiv_control),
    geom = "errorbar",
    position = position_dodge(0.9)
  ) +
  geom_boxplot(
    aes(x = Treatment_positiv_control, y = Prportion_of_Hatched_wasps,
        fill = Treatment_positiv_control),
    position = position_dodge(0.9),
    width = 0.8
  ) +
  stat_summary(
    aes(x = Treatment_positiv_control, y = Prportion_of_Hatched_wasps,
        fill = Treatment_positiv_control),
    position = position_dodge(0.9),
    fun.y = mean,
    geom = "point",
    shape = 5,
    size = 4
  ) +
  facet_grid( ~ Soil_type,
              labeller = labeller(Soil_type = soil_type.names)) +
  ylim(0, 1) +
  theme_wasp() +
  ggplot2::theme(
    legend.position = "none",
    axis.title.x = element_blank(),
    axis.text.x = element_blank(),
    axis.ticks.x = element_blank()
  ) +

  ggplot2::labs(y = expression(bold(paste(
    "Proportion hatched wasps"
  ))))
plot2_pupation_depth48
```

```
pdf("boxplot_hatched_wasp.pdf",
    width = 7,
    height = 4)
print(plot2_pupation_depth48)
dev.off()
```

Pupation depth to hatched wasps (Figure 3)

```
#Regroup Soil type
Daten_rep_test_regroup <- Daten_rep_test
Daten_rep_test_regroup$Soil_type <-
  factor(Daten_rep_test_regroup$Soil_type,
         levels = c("Loamy sand", "Loam", "Clay"))
# New facet label names for Soil type
soil_type.names <- c("L. sand", "Loam", "Clay")
names(soil_type.names) <- c("Loamy sand", "Loam", "Clay")
# Plot
plot2_pupation_depth24 <- ggplot(data = Daten_rep_test_regroup) +
  stat_boxplot(
    aes(x = Pupation_depth, y = Prportion_of_Hatched_wasps,
        fill = Pupation_depth),
    geom = "errorbar",
    position = position_dodge(0.9)
  ) +
  geom_boxplot(
    aes(x = Pupation_depth, y = Prportion_of_Hatched_wasps,
        fill = Pupation_depth),
    position = position_dodge(0.9),
    width = 0.8
  ) +
  stat_summary(
    aes(x = Pupation_depth, y = Prportion_of_Hatched_wasps,
        fill = Pupation_depth),
    position = position_dodge(0.9),
    fun.y = mean,
    geom = "point",
    shape = 5,
    size = 4
  ) +
  facet_grid( ~ Soil_type,
              labeller = labeller(Soil_type = soil_type.names)) +
  ylim(0, 1) +
  theme_wasp() +
  ggplot2::theme(legend.position = "none",
                 axis.title.x = element_blank())+
  ggplot2::labs(y = expression(bold(paste(
                   "Proportion hatched wasps"
                 ))))
                 plot2_pupation_depth24
```

```
pdf("boxplot_hatched_wasp_depth.pdf",
    width = 8,
    height = 4)
print(plot2_pupation_depth24)
dev.off()
```
