# Supplementary material for: Below ground efficiency of a parasitic wasp for Drosophila suzukii biocontrol in different soil types
Source: Sci Rep. 2022 Jun 1;12:9130. doi: 10.1038/s41598-022-12993-w (PMC9160073; doi:10.1038/s41598-022-12993-w)
Supplement: Supplementary file 3 — Supplementary Tables. [file 41598_2022_12993_MOESM3_ESM.pdf]

1 Supplementary

Article title: **Below ground efficiency of a parasitic wasp for *Drosophila suzukii* biocontrol in different soil types**

Journal: Journal of Pest Science

Author:

*Benedikt J. M. Häußling

Department of Evolutionary Animal Ecology, Bayreuth University, Bayreuth, Germany

ORCID: 0000-0003-1737-6824

Melinda Mautner

Department of Evolutionary Animal Ecology, Bayreuth University, Bayreuth, Germany

Johannes Stökl

Department of Evolutionary Animal Ecology, Bayreuth University, Bayreuth, Germany

ORCID: 0000-0002-6471-434X

*Corresponding author: Benedikt.Haeussling@uni-bayreuth.de

Table 1: Effect of time wasp added on hatched fly – Generalised linear mixed effect model (family = binomial, link = logit, random factors: "Repetition/Pupaenumber", "Percent pupated", "Mean Temperature") output quantifying the effect of time wasp added, pupation depth, soil type and the treatment (with wasp / without wasp) on the hatching rate of the fly *D. suzukii.*

Predictor	χ^2	*df*	*p*-value
Time wasp added	0.25	1	0.62
Soil type	5.07	2	0.08
Pupation depth	7.96	1	**0.005**
Treatment	2.56	1	0.11

Table 2: Effect of time wasp was added on hatched wasps – Generalised linear mixed effect model (family = binomial, link = logit, random factors: "Repetition/Pupaenumber", "Percent Pupated") output quantifying the effect of time wasp added, pupation depth and soil type, on the hatching rate of the wasp *T. drosophilae*.

Predictor	χ^2	*df*	*p*-value
Time wasp added	0.36	1	0.55
Soil type	0.75	2	0.69
Pupation depth	2.57	1	0.11

Table 3: Physical characteristics of standard soils according to GLP (LUFA, Speyer)

Particle size distribution (mm) according to USDA (%)	Loamy Sand	Loam	Clay
**<0.002**	3.9 ± 0.8	24.5 ± 1.8	**41.9** ± 2.7
**0.002 - 0.05**	8.7 ± 1.1	42.9 ± 1.35	35.1 ± 0.5
**0.05 - 2.0**	87.5 ± 1.3	32.6 ± 2.0	23.0 ± 2.4
**Maximum water holding capacity (g/100g)**	31.4 ± 2.9	45.6 ± 2.7	41.4 ± 1.5
**Weight per volume (g/1000ml)**	1435 ±53	1206 ± 58	1291 ± 47
